# Case Report and Literature Analysis: Guillain-Barré Syndrome With Delayed Unilateral Facial Palsy

**DOI:** 10.3389/fneur.2021.658266

**Published:** 2021-03-31

**Authors:** Xuanyu Huang, Ziwei Lan, Yajing Zhan, Zhiping Hu

**Affiliations:** Department of Neurology, The Second Xiangya Hospital, Central South University, Changsha, China

**Keywords:** Guillain-Barré syndrome, delayed unilateral facial palsy, threshold, anti-ganglioside antibodies, asymmetric, retreatment

## Abstract

Guillain-Barré syndrome (GBS) is an acute inflammatory polyradiculoneuropathy in which most patients have cranial nerve involvement, with facial nerve involvement being the most common. However, delayed facial palsy (DFP) with asymmetric facial palsy is a rare manifestation of GBS, and the mechanism is unclear. We report a case of GBS combined with delayed unilateral facial palsy and review previously reported cases of GBS combined with DFP. A total of 28 cases of GBS with DFP, including the case in this report, were included in this study. The occurrence of DFP may be related to early subclinical demyelination of the facial nerve, the blood-nerve barrier of the facial nerve, facial movement, and descending reversible paralysis. The occurrence of unilateral facial palsy may be related to *Campylobacter jejuni*, specific anti-ganglioside antibodies, and the site of central nervous system anatomical involvement. There is no evidence that immunotherapy is related to the shortening of DFP course and improving patients' prognosis.

## Introduction

Guillain-Barré syndrome (GBS) is an autoimmune-mediated acute inflammatory polyradiculo-neuropathy with symptoms peaking at about 2 weeks and a monophasic self-limiting course involving the spinal nerve roots, peripheral nerves, and cranial nerves. Cranial nerve involvement is most common with bilateral facial nerve palsy, rarely with unilateral involvement, and facial palsy mostly occurs in the early stage of the disease (1, 2). Delayed facial palsy (DFP) is facial palsy that occurs after other neurological signs have peaked or after symptoms have begun to improve, with an incidence of only 6% (3). Here we report on a case of a 54-years-old man who presented with both lower limb pain and weakness as the first clinical manifestation. The patient developed facial palsy after other neurological symptoms have reached nadir. Based on clinical manifestations, the same damage type of facial nerve and limb nerve as suggested by electrophysiological examination, and good treatment response to intravenous immunoglobulin (IVIG), the patient was diagnosed with GBS with delayed unilateral facial palsy. Further, we review all previous GBS cases combined with DFP, summarizing the clinical features, examination results, and treatment prognosis of such patients. Our study included a total of seven articles (3–9), with a total of 28 cases, including 14 GBS, 13 MFS, and one MFS/GBS overlapping case. Cases that met the criteria are listed in Table 1. Our purpose was to explore the mechanism of DFP and asymmetric facial palsy in GBS and clarify the main points of diagnosis and treatment options.

**Table 1:** Review all reported GBS/MFS with DFP cases.

				**Course time (days)**								
	**Diagnosis**	**Age/sex**	**Symmetry of FP**	**Nadir**	**Appearance of FP**	**Resolution of FP**	**Course time of FP**	**Course time of GBS/MFS**	**Retreatment**	**Antibodies**	**MRI**	**Pathogen testing**
Case 1	GBS	54/M	-	7	23	49	26	Incomplete resolution	IVIG	-	-	NA
**Fisher** **(****4****)**												
Case 2	MFS	45/M	-	5	13	42	29	-	NA	-	NA	NA
**Chida et al**. **(****5****)**												
Case 3	MFS	65/M	+	8	17	45	28	85	IAP	GQ1b	-	NA
Case 4	MFS	60/M	+	4	12	36	24	126	MP	GQ1b	NA	NA
**Tatsumoto et al**. **(****3****)**												
Case 5	GBS	55/M	-	5	14	29	15	38	-	GQ1b	NA	NA
Case 6	MFS	32/M	NA	6	10	NA	NA	NA	-	NA	NA	NA
Case 7	MFS	18/M	NA	6	7	NA	NA	NA	-	NA	NA	NA
Case 8	MFS	29/F	NA	10	11	NA	NA	NA	-	NA	NA	NA
Case 9	MFS	35/M	NA	5	15	NA	NA	NA	-	NA	NA	NA
Case 10	GBS	79/F	NA	6	7	NA	NA	NA	-	NA	NA	NA
Case 11	GBS	29/M	NA	6	14	NA	NA	NA	-	NA	NA	NA
Case 12	GBS	81/F	NA	7	9	NA	NA	NA	-	NA	NA	NA
Case 13	GBS	55/F	NA	9	30	NA	NA	NA	-	NA	NA	NA
Case 14	GBS	15/M	NA	9	10	NA	NA	NA	-	NA	NA	NA
Case 15	GBS	25/F	NA	10	14	NA	NA	NA	-	NA	NA	NA
Case 16	GBS	55/M	NA	11	16	NA	NA	NA	-	NA	NA	NA
Case 17	GBS	68/M	NA	6	7	NA	NA	NA	-	NA	NA	NA
Case 18	GBS	31/M	NA	4	12	NA	NA	NA	-	NA	NA	NA
**Course time (days)**												
Case 19	GBS	35/M	NA	6	15	NA	NA	NA	-	NA	NA	NA
Case 20	GBS	67/M	NA	3	10	NA	NA	NA	-	NA	NA	NA
Case 21	GBS	32/F	NA	6	7	NA	NA	NA	-	NA	NA	NA
**Liu et al**. **(****6****)**												
Case 22	MFS	40/M	+	9	10	18	8	18	MP	GM1	-	NA
**Yamamoto et al**. **(****9****)**												
Case 23	MFS	55/M	-	11	16	42	26	Incomplete resolution	VCV	GQ1b	-	-
**Tan et al**. **(****7****)**												
Case 24	MFS	52/M	-	9	14	66	52	66	-	-	NA	NA
Case 25	MFS	45/M	+	6	12	35	23	78	PE	GQ1b, GT1a, GD1b	NA	NA
Case 26	MFS	21/F	+	4	8	64	56	100	-	GQ1b, GT1a	NA	NA
Case 27	MFS/GBS	57/F	-	6	14	43	29	44	-	GQ1b, GT1a	NA	NA
**Umekawa et al**. **(****8****)**												
Case 28	MFS	47/M	+	7	14	43	29	NA	-	GQ1b, GT1a, Ga1NAc, GD1a	+	-

*M, male; F, female; GBS, guillain-barre syndrome; MFS, miller fisher syndrome; DFP, delayed facial palsy; MRI, magnetic resonance imaging; NA, not available; FP, facial palsy; C.jejuni, campylobacter jejuni; IVIG, intravenous immunoglobulin; IAP, immunoadsorbent plasma exchange; MP, methylprednisolone; PE, plasma exchange; VCV, valacyclovir*.

## Case Description

A 54-year-old male with previous physical fitness was admitted to the hospital with “both lower limb pain and weakness for 1 month, and right facial palsy for 1 week.” The patient started with weakness in both lower limbs and pain in the buttocks, the back of both thighs, calves, and feet. On the 7th day of onset, his loss of muscle strength in both lower limbs peaked, and his muscle strength began to gradually recover. Nervous system physical examination revealed incomplete closure of the right eye, shallowing of the right frontal lines and nasolabial folds, deviation of the mouth angle to the left, and normal function of the remaining cranial nerves. The power was grade 4/5 in the lower limbs and grade 5/5 in the upper limbs. Deep tendon reflexes were absent in all limbs. Sensory system examination showed that the pain of all toes was decreased and the rest were normal. Pathological reflex examination was negative. Laboratory test results indicated that routine blood, liver, and kidney function, electrolytes, and blood sugar tests all returned normal. Brain magnetic resonance imaging (MRI) revealed no abnormalities. Cerebrospinal fluid results suggested protein cell separation (cerebrospinal fluid protein 868.46 mg/L, total cell count 68 × 10^6^/L, white blood cell count 18 × 10^6^/L). Western blot analysis of serum and erebrospinal fluid anti-ganglioside antibodies (including anti-sulfatide, anti-GM1, anti-GM2, anti-GM3, anti-GM4, anti-GD1a, anti-GD1b, anti-GD2, anti-GD3, anti-GT1a, anti-GT1b, and anti-GQ1b antibodies), cell-based assay method to detect serum and cerebrospinal fluid central nervous system (CNS) demyelinating disease antibodies (including AQP4 antibody, anti-MOG antibody, anti-MBP antibody), and serum and cerebrospinal fluid IgG oligoclonal zone analysis results were negative. Complete serum and cerebrospinal fluid virus tests showed herpes simplex virus type 1 IgG (+). The latency and conduction velocity of the deep tibial and fibula motor nerve conduction velocity (NCV) were normal bilaterally, and the amplitude of the compound muscle action potential (CMAP) was reduced. A sensory nerve conduction study showed that the CMAP amplitude of the sural sensory nerve was reduced. F wave waveform was discrete on the right tibial nerve. The NCV of facial movement showed that the amplitude of the right cheek branch, cheekbone branch, and temporal lobe branch was reduced, and the conduction was normal. Blink reflex results showed that R1 and R2 on the right side were later than on the left side and had a lower amplitude than on the left side. The conclusion showed that the right facial nerve and lower limb nerves were demyelinated, accompanied by polyneuropathy and secondary axonal degeneration. The patient was diagnosed with GBS with unilateral DFP. On the 38th day of onset, the patient was administered IVIG 0.4 g/kg/day for five consecutive days. On the 49th day of onset, the patient's facial palsy completely disappeared, and the pain and weakness of the lower extremities and numbness of the feet partially resolved. After 12 months of follow-up after discharge, the patient still had mild pain in both lower extremities.

## Discussion

We report a rare case of GBS with delayed unilateral facial palsy. Unlike the symmetrical facial palsy and the typical monophasic course of GBS patients (10), this patient developed delayed unilateral facial palsy after recovery of limb muscle strength, and the results of the electrophysiological examination suggested the same type of facial nerve and limb nerve damage, and a diagnosis of GBS combined with DFP was considered. After treatment with IVIG, the patient's facial palsy rapidly improved, further confirming that delayed onset facial palsy is still part of the GBS course. As a variant of GBS, MFS usually involves ophthalmoparesis, ataxia, and areflexia as the main symptoms, without limb weakness. A positive anti-GQ1b antibody is helpful for the diagnosis (11). Although both can be combined with facial palsy, MFS rarely recurs after the condition improves. Even if relapse occurs, the interval is very long (12). Based on our literature review, the ratio of GBS combined with DFS is consistent with that of MFS (3); therefore, we presume that DFS is a delayed type that may be secondary to multiple GBS variants. If facial palsy occurs in the above situation, GBS disorder and the relative disease are priorities for diagnosis.

So far, few cases of GBS combined with DFP have been reported. Only 28 cases in seven studies have been described (including our case, listed in Table 1) (3–9). Patients' age range was 15–81 years (mean ± SD: 45.44 ± 17.71), 20 (71.4%) were male, and 8 (28.6%) were female, with a male to female ratio of 5:2. Twenty-one out of 28 patients (77.8%) had a history of antecedent illness; the upper respiratory was the main cause, followed by diarrhea. Pathogen testing was completed in only two cases, and both were negative. The neurological level of all patients peaked within 3–10 days (mean ± SD: 6.82 ± 2.20). DFP occurred 7–30 days after onset (median: 12 days). Fifteen of 15/28 patients (53.6%) had bilateral facial palsy, and 13/28 patients (46.4%) had unilateral facial palsy. Among the 16 patients reported by Tatsumoto (3), seven had unilateral facial palsy and nine had bilateral facial palsy. We can only obtain detailed symptom data of 16 patients from all articles, of which 14/16 patients (87.5%) had symptoms of cranial nerve involvement other than the facial nerve, such as ophthalmoplegia and bulbar palsy. This included 13 MFS and one MFS/GBS patient, and 2/16 patients (12.5%) had facial nerve involvement alone. Detailed cerebrospinal fluid data were available from all articles for 12 patients, and 9/12 patients (75.0%) showed protein cell separation. Fifteen out of 28 patients (53.6%) were positive for anti-ganglioside antibodies, and multiple antibodies could be positive in the same patient at the same time. Of the 15 patients, 11 tested positive for anti-GQ1b antibody, 4/15 patients tested positive for anti-GT1a antibody, of which 4/16 patients reported by Tatsumoto (3) tested positive for anti-GQ1b antibody, and two patients tested positive for anti-GM1, GM1b, GD1a, or GalNAc-GD1a antibody. Twenty-two out of 28 patients (78.6%) received immunotherapy after admission, of which 11/22 patients (50.0%) received IVIG treatment, and 5/22 patients (22.7%) received IVIG combined with methylprednisone treatment, 3/22 patients (13.6%) received plasma exchange (PE) treatment, 2/22 patients (9.1%) received immunoadsorbent plasma exchange (IAP) treatment, and 1/22 patients (4.5%) received immunoadsorption therapy. Six out of 28 patients (21.4%) received specific treatment after the onset of DFP, of which 2/6 patients (33.3%) were treated with methylprednisone, one patient (14.3%) developed DFP during treatment with PE and IAP and continued treatment, and one patient (14.3%) received IVIG and valacyclovir. The course of facial palsy was roughly 3 weeks in all 16 patients reported by Tatsumoto (3), regardless of whether they received retreatment after the onset of DFP. In addition, 6/28 patients (21.4%) did not receive any treatment during the entire course of the disease. We were able to obtain complete prognostic data from 10 patients, with all symptoms resolved in a total of eight patients, including four who did not receive retreatment after the onset of DFP, except for one patient who remained with mild bilateral abduction limitation and diplopia after treatment with IVIG and valacyclovir, and one patient who remained with mild pain in both lower extremities after treatment with IVIG. Whether the patient received retreatment after the onset of DFP did not appear to affect the course of facial palsy or the prognosis. However, we still wondered, “how does delayed unilateral facial palsy occur and what is its possible pathogenesis?” For this purpose, we launched a careful literature analysis.

In 2004, Vucic conducted a retrospective analysis of the clinical data of 38 early Acute inflammatory demyelinating polyneuropathy (AIDP) patients. Among them, the blink reflex results of 16 patients suggested that the ipsilateral R1 and R2 and the contralateral R2 were prolonged or disappeared, indicating that the facial nerve's early demyelination changes, but only 11 patients developed facial palsy (13). Similar results were obtained in a study by Wali (14). There were 17 patients with abnormal blink reflex results without facial palsy. These findings confirm that early facial neuropathy in GBS patients may be subclinical. At the same time, some researchers suggested that the progression pattern of “descending reversible paralysis” in the course of MFS leads to the emergence of DFP (15). Among 11 patients with MFS combined with DFP, nine of the patients' facial palsy symptoms appeared after the upper cranial nerves (III, IV, and VI) involved. The authors speculate that the pathophysiology of MFS may include this pattern, but it cannot explain the group of GBS patients with lower cranial nerve involvement as the first symptom and single cranial nerve involvement. In addition, in a study on proximal conduction abnormalities of the facial nerve in MFS patients, subclinical demyelinating lesions of the proximal facial nerve were detected in all three MFS patients (16). Therefore, we speculate that in some patients with GBS and DFP, the lesions of the proximal facial nerve segment may be subclinical demyelination and conduction block in the early stage. As the disease progresses, the lesion reaches a “threshold,” and the time taken to reach the threshold determines the timing of facial palsy, and this process of development leads to the emergence of “delayed onset.” Therefore, we suggest that the atypical monophasic course of GBS combined with DFP patients may be related to early subclinical demyelination of the facial nerve, the blood-nerve barrier of the facial nerve, facial movements, and the pattern of “descending reversible paralysis.”

The positive rate of anti-ganglioside antibodies in typical GBS patients is <33.3%(17), the positive rate of anti-GQ1b antibodies in patients with MFS is 80–90% (11), and the positive rate of antibodies in GBS combined with DFP is between the two (53.6%), mainly with anti-GQ1b and anti-GT1a antibodies. Therefore, we speculate that anti-ganglioside antibodies may be involved in the occurrence of DFP. In a review of previous literature, Fan et al. found that GBS patients presenting with facial palsy had a high rate of anti-ganglioside antibody IgM positivity, including 40% for anti-GM1 and GM2 antibodies and 33.3% for anti-GM3 antibodies, while the rate of positivity for other antibodies was extremely low. Therefore, they proposed that anti-ganglioside antibodies GM1, GM2, and GM3 are associated with the appearance of facial palsy (17). Greco reported a case of recurrent facial palsy in a child with elevated serum anti-GQ1b IgG and IgM titers, which decreased as clinical symptoms improved, thus suggesting that anti-GQ1b antibodies are associated with recurrent facial palsy (18). In another study, more than half of the patients with positive anti-GT1a IgG antibodies developed facial palsy. The authors speculated that patients with positive anti-GT1a IgG usually have multiple cranial nerve involvement, including the facial nerve (19). However, the mechanism of action of these antibodies in DFP still needs further research. In fact, the high positive rate of anti-GQ1b, anti-GT1a, and other antibodies in GBS patients with DFP is not directly related to the expression of gangliosides on the facial nerve. For example, GQ1b is abundantly distributed on nerves II, III, IV, and VI but less on the facial nerve and other nerves (20). Combining statistical data analysis, we propose the following hypothesis: there is more GQ1b on the facial nerve in GBS patients with combined DFP than in those with classic GBS. This remains to be confirmed in future clinical studies. It is worth noting that of the 28 cases of GBS combined with DFP summarized in this article, 13 cases still had negative anti-ganglioside antibody tests. We speculate that there may also be antibodies involved in the occurrence of DFP that have not yet been discovered. Combined with the clinical characteristics of DFP, we found that the sequence of the antibody's effects on different receptors may be affected by the abundance of blood supply and the blood-nerve barrier. For example, the blood supply of the extraocular muscle capillaries is abundant, and toxins and antibodies in the circulation preferentially enter this site, so that conduction abnormalities appear in the early stage of the disease. However, the blood-nerve barrier of the facial nerve is relatively intact, and antibodies to the facial nerve reach the target later, resulting in delayed facial nerve involvement. Previous literature suggests that physical factors, such as limb movement and body posture, may affect the initial distribution of weakness symptoms. For example, the arms and legs are in a state of exercise for a long time, and blood flow increases at certain critical moments when inflammatory cells or humoral factors enter the nerves, which leads to the first appearance of fatigue symptoms (21). For some patients with relatively less facial muscle activity than limbs, this may be one of the reasons for DFP.

GBS usually presents with widespread and symmetrical muscle weakness, often involving the bilateral extremities and accompanied by hyporeflexia/absence of reflexes (22). In contrast, the case reported here presented with asymmetric bilateral facial nerve involvement, a manifestation that occurred in only 4.9% of GBS (1). The common clinical unilateral facial palsy is easily misdiagnosed as Bell's palsy. The main treatment to promote the recovery of facial palsy in Bell's palsy patients is corticosteroids, but it has no effect on improving the prognosis of GBS and may even have adverse effects (23). Therefore, the distinction between the two is important. Studies have shown that more than 57% of patients with Bell's palsy have pathological enhancement of the facial nerve (24). Kinoshita compared head-enhanced MRI of 125 patients with Bell's palsy to 300 controls without facial nerve disease and showed that 67 and 43% of Bell's palsy patients had distal intrameatal and facial nerve labyrinthine segment enhancement, respectively, compared to 0% of the controls (25). Head-enhanced MRI of patients with GBS with facial palsy rarely shows facial nerve enhancement, and the enhancement segments are irregular (26–29). Therefore, when DFP appears in GBS patients, enhanced head MRI can be performed to help identify Bell's palsy. A study reported a patient with GBS with marked asymmetry in clinical symptoms and electrophysiological manifestations, whose serologic testing suggested a recent *Campylobacter jejuni* infection and anti-ganglioside antibody testing suggested a significantly elevated titer of anti-GM1 IgG. Therefore, the researchers concluded that this asymmetry was associated with *C. jejuni* infection and high titers of anti-GM1 IgG (30). Campylobacter jejuni is the most common pathogenic microorganism that mediates GBS/MFS autoimmunity (10). Autoreactive immunoglobulin G1 (IgG1) is a common antibody subtype after human Campylobacter jejuni infection, and the IgG1 titer is related to the severity of GBS and prognosis (31). Malik et al. showed that IgG1 can cross-react with peripheral gangliosides GM1 and GD1a to induce the production of anti-ganglioside antibodies (32). Campylobacter jejuni in MFS can also induce autoimmunity to produce anti-GQ1b antibodies (10). In this study, only two patients underwent the pathogenic test and were negative. Compared with patients with classic GBS/MFS, patients with DFP have a lower positive rate of Campylobacter jejuni, which may be related to the lack of attention to pathogenic examination by previous researchers. Therefore, we suggest that in the future, more attention should be paid to the pathogenic examination of patients with GBS/MFS with DFP to better study the role of Campylobacter jejuni in the occurrence of DFP and asymmetric facial palsy. Osaki reported a case of a significantly asymmetric pharyngeal-cervical-brachial variant GBS. The anti-GT1a antibody titer increased in parallel with the clinical symptoms. The authors proposed that the evaluation of anti-ganglioside antibodies (including anti-GT1a IgG antibodies) should help in diagnosing GBS with asymmetric involvement (33). In addition, an autopsy study of GBS patients confirmed that GBS can affect the CNS, which involves axons with secondary myelin damage, microglial activation, and inflammatory infiltration (34). Some researchers have suggested that CNS involvement can lead to asymmetric symptoms and signs in patients with GBS (35). Thus, *C. jejuni* infection, anti-ganglioside antibodies, and asymmetric CNS involvement may all contribute to the asymmetric distribution of GBS symptoms.

To date, there have been no systematic studies on the treatment of patients with GBS combined with DFP. In the cases reviewed in this article, 78.6% of the patients received specific treatment and achieved complete remission of symptoms. Therefore, the recommended treatment options for such patients, IVIG and PE, are still preferred (22). However, after summarizing the cases, we also found that regardless of whether the patient received retreatment after the occurrence of DFP, the course of facial palsy was about 3 weeks. Currently, there is no evidence that immunotherapy can shorten the course of facial palsy and improve the prognosis.

## Conclusion

In this study, we reported a case of GBS with rare features of delayed unilateral facial palsy at the same time. The facial and peripheral nerves had the same electrophysiological changes. We reviewed all previously reported cases of GBS combined with DFP and concluded that the occurrence of DFP may be related to the early subclinical demyelination of the facial nerve, the blood-nerve barrier of the facial nerve, facial movement, and descending reversible paralysis, and the occurrence of unilateral paralysis may be related to *C. jejuni*, specific anti-ganglioside antibodies, and CNS anatomical involvement. Although IVIG and PE can be used for treatment, there is no evidence that immunotherapy is related to the shortening of the DFP course and improving patients' prognosis. We hope that this study can provide references for clinical diagnosis and treatment, but the pathogenic mechanisms of such diseases need to be verified by larger studies.

## Data Availability Statement

The original contributions generated for the study are included in the article/supplementary material, further inquiries can be directed to the corresponding author/s.

## Ethics Statement

The studies involving human participants were reviewed and approved by the Ethics Committee of The Second Xiangya Hospital. The written consent to publish this information has been obtained from the study patient. The patients/participants provided their written informed consent to participate in this study. Written informed consent was obtained from the individual(s) for the publication of any potentially identifiable images or data included in this article.

## Author Contributions

XH conducted the literature review and drafted the manuscript. ZL made substantial contributions to conception and interpretation of data. ZH and YZ were involved in revising the manuscript critically and have given final approval of the version to be published. All authors read and approved the manuscript.

## Conflict of Interest

The authors declare that the research was conducted in the absence of any commercial or financial relationships that could be construed as a potential conflict of interest.
